# Olaparib increases the therapeutic index of hemithoracic irradiation compared with hemithoracic irradiation alone in a mouse lung cancer model

**DOI:** 10.1038/s41416-021-01296-y

**Published:** 2021-03-19

**Authors:** Yanyan Jiang, Jennifer Martin, Maryam Alkadhimi, Kay Shigemori, Paul Kinchesh, Stuart Gilchrist, Veerle Kersemans, Sean Smart, James M. Thompson, Mark A. Hill, Mark J. O’Connor, Barry R. Davies, Anderson J. Ryan

**Affiliations:** 1 CRUK & MRC Oxford Institute for Radiation Oncology, Department of Oncology, University of Oxford, Oxford, UK; 2 Early Oncology, AstraZeneca, Cambridge, UK

**Keywords:** Cancer models, Radiotherapy, Non-small-cell lung cancer

## Abstract

**Background:**

The radiosensitising effect of the poly(ADP-ribose) polymerase inhibitor olaparib on tumours has been reported. However, its effect on normal tissues in combination with radiation has not been well studied. Herein, we investigated the therapeutic index of olaparib combined with hemithoracic radiation in a urethane-induced mouse lung cancer model.

**Methods:**

To assess tolerability, A/J mice were treated with olaparib plus whole thorax radiation (13 Gy), body weight changes were monitored and normal tissue effects were assessed by histology. In anti-tumour (intervention) studies, A/J mice were injected with urethane to induce lung tumours, and were then treated with olaparib alone, left thorax radiation alone or the combination of olaparib plus left thorax radiation at 8 weeks (early intervention) or 18 weeks (late intervention) after urethane injection. Anti-tumour efficacy and normal tissue effects were assessed by visual inspection, magnetic resonance imaging and histology.

**Results:**

Enhanced body weight loss and oesophageal toxicity were observed when olaparib was combined with whole thorax but not hemithorax radiation. In both the early and late intervention studies, olaparib increased the anti-tumour effects of hemithoracic irradiation without increasing lung toxicity.

**Conclusions:**

The addition of olaparib increased the therapeutic index of hemithoracic radiation in a mouse model of lung cancer.

## Background

Radiation therapy is an important local treatment for many cancers, including lung cancer. However, despite technical advances in radiotherapy, anti-tumour efficacy remains suboptimal in part due to dose-limiting normal tissue toxicity. Therefore, there is an urgent need for agents that can increase the therapeutic index of radiotherapy by increasing the anti-tumour effects without increasing normal tissue toxicity.

Radiation generates reactive oxygen species that damage DNA, causing mostly single-strand breaks (SSBs) that, if not repaired, will lead to lethal double-strand breaks. Poly(ADP-ribose) polymerase 1 (PARP-1) is able to detect radiation-induced DNA SSBs, and then recruit DNA repair proteins to facilitate DNA repair.^1^ Following radiation, inhibition of PARP-1 inhibits SSB DNA repair, which can inhibit DNA synthesis leading to collapsed replication forks, increased DNA double-strand breaks and the subsequent cell death.^2^ PARP inhibition has been shown to enhance the effects of radiotherapy in various pre-clinical tumour models including lung cancer,^3–6^ breast cancer,^4^ prostate cancer and colon cancer.^7^

Olaparib (AZD2281) is a potent oral PARP-1 inhibitor.^8^ Despite a variety of pre-clinical studies showing increased sensitivity of tumours to radiation by olaparib,^5,9–11^ the effect of olaparib on the response of normal tissues to radiation has been little studied in vivo. Our recent study has shown that although PARP inhibition enhanced the anti-tumour response to radiation in human lung cancer xenograft models, it also increased oesophageal toxicity induced by whole thoracic radiation in C57BL6 mice, leading to body weight loss, suggesting that PARP inhibitors can augment radiation toxicity to normal tissue.^12^ However, in that study, efficacy and normal tissue effects were assessed in different animal models. Furthermore, although olaparib has demonstrated tolerability in clinical trials,^13^ phase 1 trials on the safety and tolerability of olaparib in combination with thoracic radiotherapy regimens in non-small cell lung cancer (NSCLC) are still ongoing.^14^

The aim of the present study was to evaluate whether combining the PARP inhibitor olaparib with thoracic radiation at different stages of tumour development can improve the therapeutic index of radiation alone in a lung cancer model. To this end, we used urethane-treated A/J mice, a chemically induced experimental model of KRAS-driven lung cancer^15^ in which tumours arise stochastically in the lung over several months, thus allowing the potential for evaluation of anti-tumour efficacy and acute and late normal tissue effects in the same model.

## Methods

All animal experiments were performed under project license 30/3395 issued by the UK Home Office after local ethical review. Female A/J mice (6–8 weeks old) were purchased from Envigo (UK) and were housed in ventilated cages in a room with 12 h dark and light cycle maintained at 22 °C and 55% humidity. Animals were monitored for clinical signs daily and weighed three times per week. Animals were allocated to treatment groups with matched mean starting weights, and, subsequently, treatment allocation was randomised between groups. Euthanasia was by an overdose of phenobarbitone followed by removal of the heart.

### Urethane-induced mouse lung cancer model

Mouse lung cancer was generated by intraperitoneal (i.p.) injection of 5% urethane to A/J mice on days 0 and 6 as previously described.^16^

### Treatment schedules

In the tolerability test, A/J mice were irradiated to the whole thorax at a single dose of 13 Gy. Mice were given vehicle (*n* = 3, 2.5% dimethyl sulfoxide/10% 2-hydroxypropyl-β-cyclodextrin) or olaparib (*n* = 3, 100 mg/kg, per os (p.o.), AstraZeneca, UK) 1 h before and 2 h after radiation (*n* = 3/group). All mice were euthanised 2 weeks after treatment. Bromodeoxyuridine (BrdU) was administered (10 mg/ml in saline, 200 μl/mouse i.p.) 1.5 h prior to euthanasia.

In an early intervention anti-tumour study, A/J mice were divided into four groups at 8 weeks after urethane injection (*n* = 3–9/group): (A) p.o. vehicle, (B) p.o. olaparib (50 mg/kg, AstraZeneca, UK), (C) 5 Gy left thorax radiation and (D) olaparib 1 h before 5 Gy left thorax radiation. All treatments were repeated five times, 3 days apart. All mice were subjected to magnetic resonance imaging (MRI) before euthanasia 24 weeks after treatment.

In a late intervention anti-tumour study, A/J mice were divided into four treatment groups 18 weeks after urethane injection (*n* = 6/group): (A) vehicle, (B) olaparib (100 mg/kg), (C) 13 Gy left thorax radiation and (D) olaparib 1 h before 13 Gy left thorax radiation. Mice were imaged by MRI prior to and at 4 and 6 weeks after treatment. All animals were euthanised immediately after the final MRI.

For the early and late intervention studies, the primary endpoint for efficacy experiments was the comparison between superficial tumour counts in radiation + vehicle versus radiation + olaparib groups. Appropriate group sizes (*n* ≥ 6) for the primary comparisons were determined assuming a large effect size (*d* = 1.6) with 80% power to reject the null hypothesis (*α* = 0.05, one-sided).

Lungs and oesophagi were collected and fixed in 10% buffered formalin at 4 °C for 24 h and then transferred to 70% ethanol. Tumours on the left and right lung surface were visually counted.

For thoracic radiation treatment, mice were anaesthetised with isoflurane and restrained in a heated lead-shielded container (6 mm thickness) with only the whole thorax or left thorax exposed. X-rays were delivered using a Gulmay medical RS320 irradiation system (Gulmay Medical Ltd, Camberley, UK) at 300 kV, 10 mA and 2.02 Gy/min. The fractionated dose schedule and single dose schedule were selected for similar biological equivalence for lung. The biologically equivalent dose for lung (*α*/*β* ratio = 3.3)^17^ was 62.9 Gy for 5 × 5 Gy and 64.2 Gy for 1 × 13 Gy. The biologically equivalent dose for tumours (*α*/*β* ratio = 10)^18^ was 37.5 Gy for 5 × 5 Gy and 29.9 Gy for 1 × 13 Gy.

### Magnetic resonance imaging

MRI was performed at 7.0 T (VNMRS, Varian Inc., CA) as previously described^19^ with a slight modification. Briefly, individual isoflurane-anaesthetised mice with respiration at 40–60 breaths/min were placed at a supine position in a custom-made, 3D printed cradle.^20^ A 45-mm-long 32 mm ID quadrature birdcage coil (Rapid Biomedical, Germany) was used for signal transmission and reception. Cardio-respiratory synchronised bSSFP (balanced steady-state free-precession) scans were operated at an isotropic resolution of 250 µm.^21^ Analogue respiration and electrocardiogram signals were processed using a custom-made gating unit to control the gating. MR images were analysed using the software ITK-SNAP.^22^

### Histology and immunohistochemistry (IHC)

Formalin-fixed oesophagus and lung samples were embedded in paraffin. Four-micrometre sections were stained with haematoxylin–eosin (H&E) for general histology and Masson’s Trichrome staining (#HT15, Sigma-Aldrich, UK) for collagen detection. The grade of lung fibrosis was scored using a modified Ashcroft scale^23^ scoring the extent of the most severe fibrosis. A single central lung slice was evaluated per animal.

BrdU and α-smooth muscle actin (α-SMA) IHC were performed using the EnVision G2 Doublestain System (Dako) according to the manufacturer’s instruction. The primary antibodies used were BrdU antibody (1:100, BD Biosciences) and α-SMA antibody (1:31,500, Sigma). Central whole lung sections were scanned and analysed using the Aperio CS scanner and ImageScope analysis software (Aperio Technologies, Oxford, UK).

### Statistical analysis

Data were expressed as mean ± SEM, and analysed using the GraphPad Prism 8.0 software (GraphPad Software Inc., USA). Two groups comparison was analysed by Student’s *t* test, and more than two groups comparison was analysed by one-way analysis of variance with Bonferroni correction. Fibrosis scores were analysed by nonparametric analysis of variance followed by Dunn’s correction. Statistical significance was defined as *P* ≤ 0.05 (two-tailed test).

## Results

### Combination of olaparib with whole thorax radiation increases oesophageal toxicity in A/J mice

**Fig. 1:**
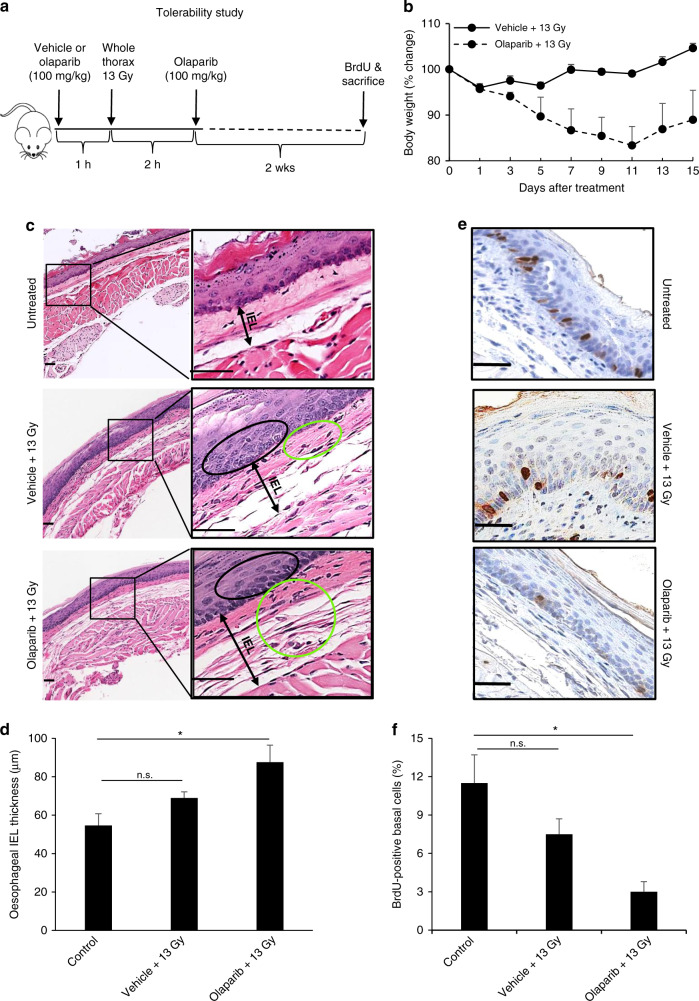
Olaparib in combination with whole thoracic radiation causes normal tissue toxicity. **a** Experimental schematic for the tolerability test: A/J mice were given vehicle or olaparib (100 mg/kg, p.o.) 1 h prior to and 2 h after 13 Gy radiation on the whole thorax. Mice were euthanised 2 weeks after the treatment and BrdU was injected 1.5 h before euthanasia. Oesophagi were collected for H&E or IHC staining. **b** Mouse body weight change was monitored and plotted over 2 weeks time after the treatment. The plot showed that olaparib combined with whole thoracic radiation caused body weight loss. **c** Representative H&E staining of oesophageal tissue. Arrows delineate IEL (inner oesophageal layer) thickness. Epithelial hyperplasia and hypertrophy were circled black. Inflammatory cell infiltration circled green. **d** Thickness of the inner oesophageal layer was measured at four representative spots of four different cross-section regions of each oesophagus (*n* = 3/group). **e** Representative BrdU staining of oesophageal tissue. **f** Quantification of BrdU+ basal cells in oesophagus epithelial layer. A minimum of 400 basal cells were counted. Graph represents mean ± SEM; *P ≤ 0.05; n.s. not significant. Scale bars: 50 μm.

A fractionated dose of 25 Gy (5 × 5 Gy) and a biologically equivalent single dose of 13 Gy^24^ were chosen for thoracic radiation in these studies as pilot studies had shown that these doses have significant anti-tumour activity in the A/J model that could potentially be increased by combination treatment. To investigate the effect of combination treatment with olaparib and thoracic radiation, we first tested the tolerability of this combination in A/J mice. Our previous studies had shown that the PARP inhibitor talazoparib induced significant oesophageal toxicity in combination with radiation in C56BL6 mice,^12^ so we first determine whether olaparib had the potential to increase oesophageal toxicity in A/J mice. We irradiated the whole thorax of mice at a single dose of 13 Gy and administered vehicle or olaparib and monitored body weight of mice over 2 weeks after the treatment (Fig. 1a). Radiation alone caused a maximum weight loss of 4.3% on day 1 after treatment, which returned to the baseline levels by day 15. In contrast, olaparib in combination with whole thorax radiation caused a maximum weight loss of 17% on day 11, with some evidence of recovery by day 15 (Fig. 1b). One of the mice in the combination treatment group lost 20% body weight and was euthanised on day 11. These data suggest that the combination of olaparib and whole thorax radiation was not well tolerated in A/J mice. To assess potential oesophageal toxicity induced by the combination treatment in A/J mice, tissue sections collected 2 weeks after treatment were stained with H&E and for BrdU-positive staining. Histopathological changes were observed in multiple oesophagus layers of the mice treated with thoracic radiation, such as hyperplasia and hypertrophy in the epithelial layer and inflammatory cell infiltration in the lamina propria and mucosa layers. Inflammatory cell infiltration was more evident in mice treated with olaparib plus radiation (Fig. 1c). Compared with untreated controls, oesophagi of mice treated with olaparib and radiation showed a significant thickening of the inner oesophageal layer (composed of lamina propria, muscularis mucosae and submucosa) (*p* < 0.05), but those treated with radiation and vehicle did not (*p* = 0.51) (Fig. 1c, d). In addition, the percentage of BrdU-positive cells in the oesophageal basal layer was reduced significantly in mice treated with olaparib and radiation compared with untreated controls (*p* < 0.05), suggesting decreased epithelial proliferation. In contrast, the percentage of BrdU-positive cells in the oesophageal basal layer was not reduced significantly in mice treated with vehicle and radiation, compared with controls (*p* = 0.42) (Fig. 1e, f). Taken together, these results suggest that olaparib combined with whole thorax radiation is less well tolerated than radiation alone and is associated with increased oesophageal toxicity, a known acute toxicity of radiation therapy.^25^

Since we showed that olaparib combined with whole thorax radiation increased oesophageal toxicity in A/J mice, we focussed our experiments on investigating the impact of olaparib only the therapeutic index of hemithorax irradiation, that is, sparing the oesophagus. In order to further mitigate potential toxicity in subsequent experiments, we reduced the dose of olaparib to 50 mg/kg given 1 h before radiation, a dose/schedule that we had previously shown to be effective in increasing the anti-tumour activity of radiation in human lung cancer xenograft models and also shown evidence of oesophageal toxicity when whole thorax irradiation was combined with 50 mg/kg olaparib in C57BL6 mice.^11,12^

### Olaparib in combination with hemithoracic radiation does not exacerbate radiation-induced body weight loss or late lung toxicity in an early intervention anti-tumour setting

**Fig. 2:**
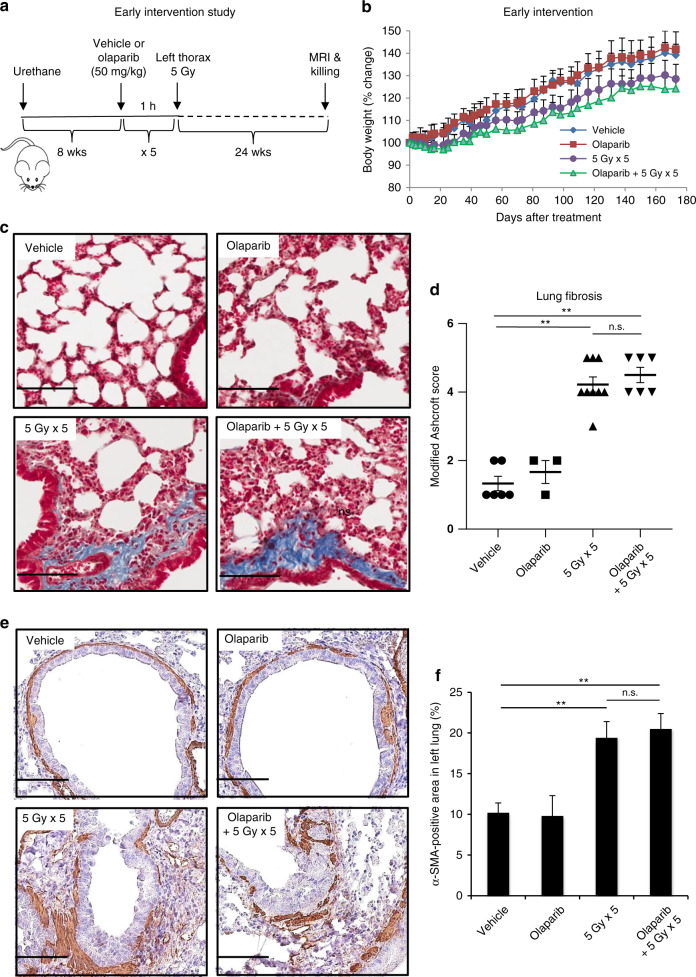
Early intervention with olaparib and hemithorax radiation does not increase radiation-induced body weight loss or lung fibrosis. **a** Experimental schematic for the early intervention study: A/J mice were i.p. injected with urethane to induce lung tumour development. After 8 weeks, mice were given olaparib (50 mg/kg, p.o.) or vehicle 1 h prior to 5 Gy hemithoracic radiation on the left side. The treatment was repeated five cycles. Mice were euthanised 24 weeks after the initiation of treatment. **b** Body weight changes of mice in four groups were recorded and plotted after treatment initiation. *n* = 3–9/group. **c** Representative Masson’s Trichrome staining of left lung tissue, with collagen fibres dyed blue. **d** Lung fibrosis was scored using the modified Ashcroft system on a scale of 0–8. Symbols represent individual animals within each treatment group. **e** α-SMA IHC staining of left lung sections. **f** Quantification of α-SMA-positive area in left lung sections. All data are expressed as mean ± SEM. **P < 0.01; n.s. not significant. Scale bars: 100 µm.

We first studied the effects of treatment at an early stage of lung tumour development (early intervention). At 8 weeks after urethane treatment, A/J mice were divided into four groups, receiving: (A) vehicle, (B) olaparib, (C) left thorax radiation (5 Gy) and (D) olaparib 1 h before left thorax radiation (5 Gy). Treatments were repeated for five cycles, and animals were euthanised 24 weeks post treatment (Fig. 2a).

After the initiation of treatment, there were no marked differences in body weight change between control and olaparib-treated groups (Fig. 2b). Compared with baseline, mice treated with hemithoracic radiation alone had a maximum mean body weight loss of 2% 2 weeks after treatment, and then had gained +29% in weight at 24 weeks. Mice treated with combination treatment lost a maximum of 3% mean body weight at 3 weeks after treatment and gained +24% body weight at 24 weeks (Fig. 2b). Although the combination treatment group had mean lower body weights than the radiation-alone group throughout the experiment (Fig. 2b), the difference was not significant, suggesting that olaparib combined with hemithoracic radiation was not significantly less well tolerated than radiation treatment alone.

We next determined whether olaparib potentiated radiation-induced late toxicity in the lung. Based on a previous report in C57BL6 mice^26^ and our preliminary studies in A/J mice, we showed moderate regional/focal lung fibrosis could be induced 24 weeks 5 × 5 Gy thoracic radiation. We, therefore, collected lungs at 24 weeks after treatment and evaluated the collagen content in the left lungs using Masson’s Trichrome stain (Fig. 2c). In vehicle- and olaparib-treated groups, the left lungs displayed isolated thickening of alveolar septa with no inflammatory infiltration. In hemithoracic radiation alone or combined with olaparib group, narrowed pulmonary alveolus cavities, thickened alveolar wall, inflammatory cell infiltration and collagen deposition were observed in the left lung interstitium (Fig. 2c), suggesting radiation-induced fibrosing alveolitis.^27^ Quantification of fibrosis revealed no significant difference between the radiation-alone group and the combination group (*p* > 0.5) (Fig. 2d), suggesting that this dose and schedule of olaparib did not exacerbate hemithoracic radiation-induced late lung toxicity in the A/J model.

Lung fibrosis was further confirmed by α-SMA IHC. α-SMA is a marker of activated fibroblasts that can produce excess collagen, resulting in pulmonary fibrosis.^28^ In the left lungs from vehicle or olaparib group, α-SMA expression was restricted to bronchial and vascular smooth muscle cells. Whereas in the left lungs exposed to radiation, upregulated α-SMA expression was found not only in bronchial and vascular smooth muscle cells but also in fibrotic foci (Fig. 2e), indicating that radiation promotes the myofibroblast activation and excessive collagen production. Although radiation significantly increased α-SMA-positive area (*p* < 0.01), there was no significant difference between radiation group and combination group (*p* > 0.5) (Fig. 2f), suggesting that the addition of olaparib did not further increase the fibroblast activation to exacerbate radiation-induced lung fibrosis.

### Early intervention with olaparib and hemithoracic radiation enhances inhibition of lung tumour development compared with hemithoracic radiation treatment alone

**Fig. 3:**
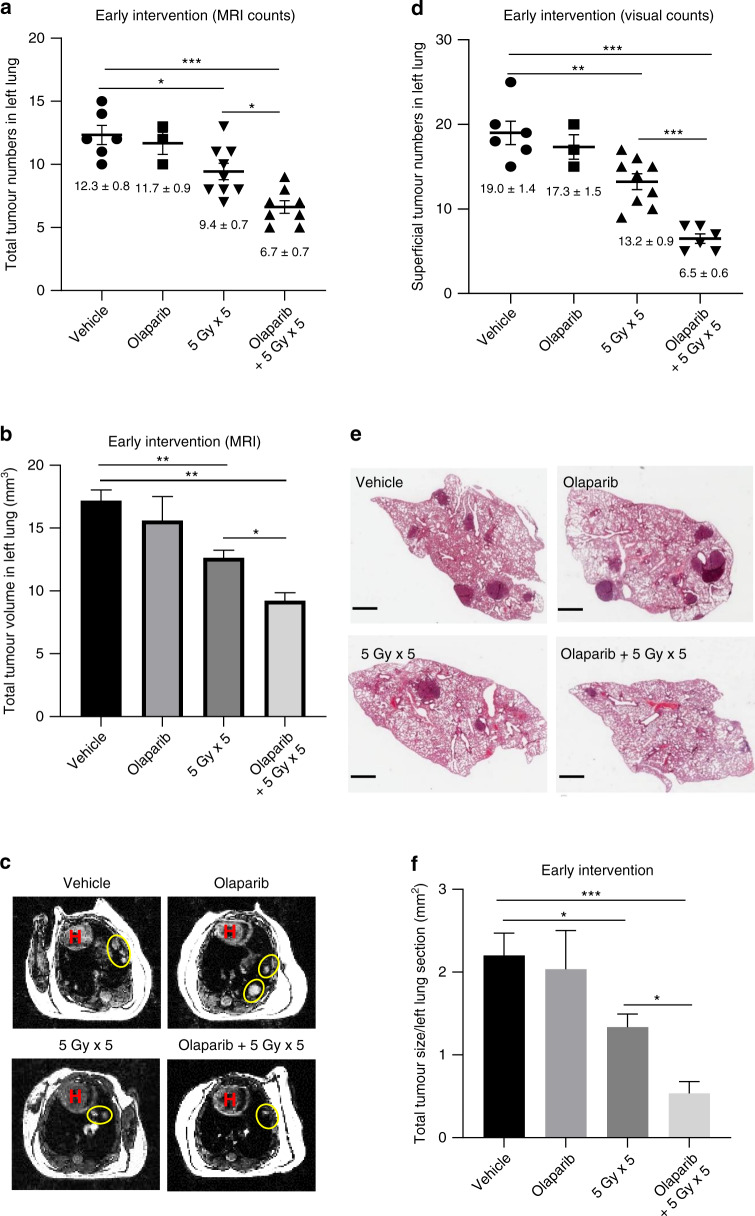
Early intervention with olaparib plus hemithoracic radiation inhibits lung tumour development. Urethane-injected A/J mice were treated with five fractions of 5 Gy left thorax radiation with or without olaparib (50 mg/kg) at 8 weeks after urethane injection (dosing as in Fig. 2). Lungs were imaged by MRI before euthanasia at 24 weeks post treatment. Lungs were collected for surface tumour count and H&E staining. **a** Quantification of total tumour number in left lungs by MRI. Symbols represent individual animals within each treatment group. **b** Quantification of total tumour volume in left lungs by MRI. **c** Representative axial MRI images of the mouse chest (H heart; tumours in the left lung were circled yellow). **d** Quantification of the tumour number on left lung surface by eye. Symbols represent individual animals within each treatment group. **e** Representative H&E staining of left lung sections. Scale bars: 1 mm. **f** Quantification of total tumour size of left lung sections in H&E images. All data are expressed as mean ± SEM. *P < 0.05, **P < 0.01 and ***P < 0.001.

Since olaparib did not increase lung toxicity of hemithoracic radiation, we next assessed whether olaparib enhanced the anti-tumour activity of hemithoracic radiation when treating at an early stage of tumour development. A/J mice were treatments 8 weeks after tumour induction by urethane (Fig. 2a). The total number and volume of the lung tumours were quantified by MRI at the end of the experiment (24 weeks post treatment). MRI measurements^19^ revealed that the total tumour number and volume in left lungs of olaparib-treated mice are comparable to those of vehicle-treated mice. Radiation alone modestly reduced tumour number by 23% (*p* < 0.05) and volume by 26% (*p* < 0.01) in left lungs compared to vehicle control (Fig. 3a–c). Olaparib combined with radiation further reduced both tumour number and volume by 23% and 20%, respectively, in irradiated left lungs (*p* < 0.05 vs radiation) (Fig. 3a–c). Interestingly, although the right lungs in the combination group were not irradiated, they displayed fewer and smaller tumours compared to those in the vehicle group (*p* < 0.05) (Supplementary Fig. S1A, B), suggesting the possibility of effects outside the radiation field. Unirradiated right lungs in the left thorax radiation group also showed decreased tumour burden but did not reach statistical significance (*p* > 0.05) (Supplementary Fig. S1A, B).

Consistent with the MRI result, radiation alone markedly reduced the number of visible surface tumours on the left lung surface (*p* < 0.01 vs the vehicle control), and addition of olaparib significantly further reduced the number of the superficial tumours (*p* < 0.0001 vs radiation alone) (Fig. 3d). The combination treatment also showed an inhibitory systemic effect on the superficial tumour counts of unirradiated right lungs compared to those in the vehicle group (*p* < 0.01) (Supplementary Fig. S1C). In addition, H&E staining of left lung sections also showed that combination treatment significantly further decreased tumour area compared with radiation alone (*p* < 0.05) (Fig. 3e, f).

Taken together, these data suggest that combination treatment with olaparib and hemithoracic radiation has a greater therapeutic index than radiation alone in this early intervention setting since there is increased anti-tumour effects without any evidence for increased lung toxicity.

### Olaparib combined with hemithoracic radiation is more effective than hemithoracic radiation alone in a late intervention anti-tumour setting

**Fig. 4:**
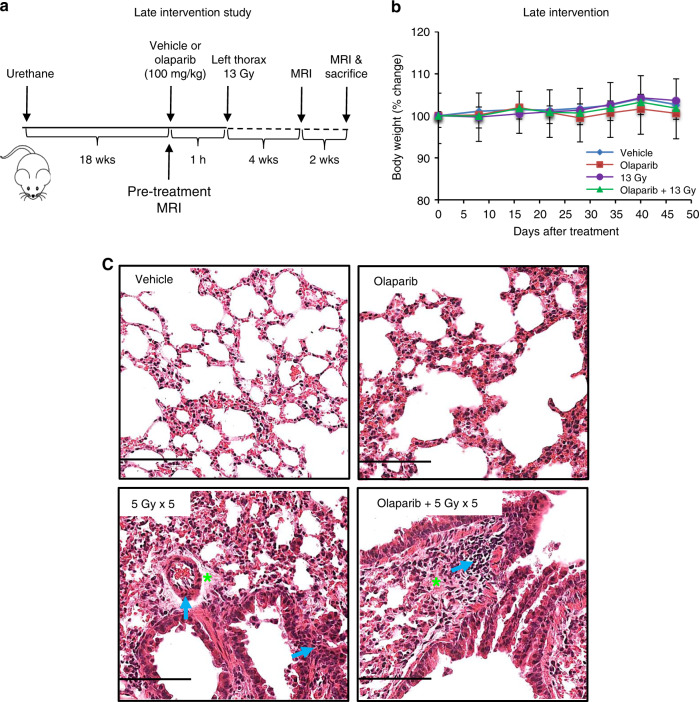
Late intervention with olaparib and hemithoracic radiation does not affect body weight or acute lung toxicity. **a** Experimental schematic for the late intervention study: 18 weeks after urethane injection, A/J mice were given olaparib (100 mg/kg, p.o.) or vehicle 1 h prior to a single dose of 13 Gy left thorax radiation. Mice were euthanised 6 weeks after treatment. MRI was performed at 0, 4 and 6 weeks after the treatment. Arrows indicate the sequence and timings of treatments. **b** Body weight changes of mice were recorded and plotted after treatment initiation. *n* = 6/group. The graph represents mean ± SEM. **c** Representative H&E-stained left lung sections. Scale bars: 100 µm. Green stars indicate interstitial oedema. Blue arrows indicate infiltration of inflammatory cells.

To determine the therapeutic index of late intervention in the A/J lung cancer model, mice were divided into four groups (Fig. 4a): (A) vehicle, (B) olaparib, (C) 13 Gy left thorax radiation and (D) olaparib 1 h before 13 Gy left thorax radiation,18 weeks after urethane induction, when tumours are well established.^19^ All animals were euthanised 6 weeks post treatment.

The late intervention combination regimen was tolerated as evidenced by no significant body weight loss in any of the treatment groups (Fig. 4b). H&E staining of histological sections of radiation-treated animals 6 weeks after treatment showed evidence of pneumonitis (thickened alveolar walls, interstitial oedema, infiltration of inflammatory cells around the vascular vessels and within the alveolar spaces) compared with controls or olaparib-treated animals (Fig. 4c). The histological changes in animals treated with olaparib plus radiation were similar to those from animals treated with radiation alone, suggesting that the addition of olaparib did not exacerbate radiation-induced inflammation.

**Fig. 5:**
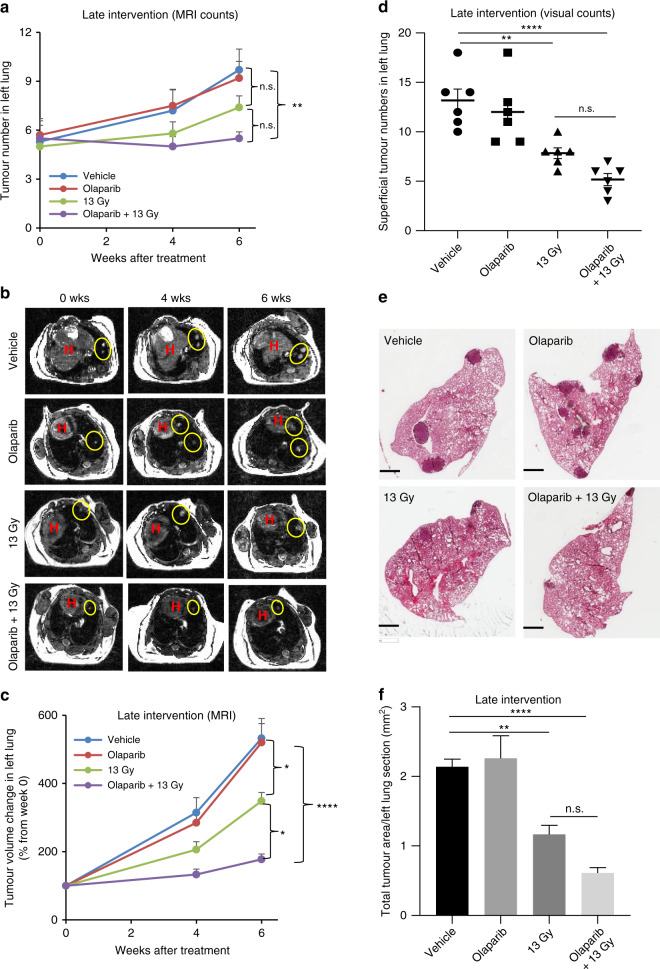
Late intervention with olaparib and hemithorax radiation inhibits lung tumour growth. Urethane-injected A/J mice were grouped (*n* = 6/group) to receive vehicle, olaparib (100 mg/kg), 13 Gy left thorax radiation alone or combined with olaparib at 18 weeks after urethane injection (dosing as in Fig. 4). Lungs were imaged by MRI at 0, 4 and 6 weeks after treatment, respectively. Lungs were collected for surface tumour count and H&E staining after the final MRI. **a** Quantification of the total tumour number in left lungs by MRI over time. **b** Representative axial MRI images of the mouse chest (H heart; tumours in the left lung were circled yellow). **c** Quantification of total tumour volume in left lungs by MRI over time. **d** Visual scoring tumour number on the left lung surface. Symbols represent individual animals within each treatment group. **e** Representative H&E-stained left lung sections. **f** Quantification of total tumour size of left lung sections in H&E images. All data are expressed as mean ± SEM. *P < 0.05, **P < 0.01 and ****P < 0.0001; n.s. not significant.

To evaluate the anti-tumour efficacy of late intervention treatments, mice were imaged by MRI before treatment, after 4 weeks and immediately before euthanasia at 6 weeks, to monitor the progression of lung tumour growth. Six weeks after treatment, detectable tumours in the left lungs of control mice showed a marked increase in number (10 ± 1 vs pretreatment 5 ± 1) (Fig. 5a) and volume (532 ± 58% vs pretreatment) over the time course of the experiment (Fig. 5b, c) in line with our previous reports for this model.^19^ Olaparib-treated mice displayed a similar increase in tumour number (9 ± 1 vs pretreatment 6 ± 1) (Fig. 5a) and volume compared with control (Fig. 5b, c). Radiation treatment alone reduced both the number and growth of left lung tumour tumours compared with controls, although only the effect on tumour growth reached statistical significance (Fig. 5a, c). Combination treatment with olaparib and radiation further reduced both tumour number compared with radiation alone (Fig. 5a, c), but again, only the effect on tumour size reached statistical significance (Fig. 5c). Similar to the early intervention treatment, the unirradiated right lungs of the combination group also showed a lower tumour burden compared to those of the vehicle control group (Supplementary Fig. S2A, B).

Consistent with the MRI analyses, there was no significant difference in the number of visible surface tumours on left lungs between vehicle- and olaparib-treated mice (Fig. 5d), suggesting that olaparib alone did not affect lung tumour growth. Whereas the irradiated left lungs showed a significant reduction of superficial tumour counts compared to the control (*p* < 0.01 for radiation alone; *p* < 0.0001 for combination treatment), and the combination treatment further significantly reduced the surface tumour number (*p* < 0.05 vs radiation alone). In addition, the unirradiated right lungs in the combination group also showed fewer surface tumours compared to those in the control group (*p* < 0.05) (Supplementary Fig. S2C).

Histological analysis of left lung central sections confirmed that hemithorax radiation modestly inhibited tumour growth compared to the vehicle control (*p* < 0.01), and olaparib combining hemithorax radiation further inhibited tumour growth (*p* < 0.0001 vs radiation alone), although the difference between the radiation group and the combination group did not reach a statistical significance (*p* = 0.193) (Fig. 5e, f).

Taken together, these results indicated that treatment of established tumours with olaparib and hemithoracic radiation enhanced the anti-tumour efficacy of radiation alone.

## Discussion

The radiosensitising effect of the PARP inhibitor olaparib on tumours has been reported in pre-clinical lung cancer models.^11,29,30^ However, the effect of olaparib and thoracic radiation on normal tissue toxicity has rarely been assessed in vivo. Here, using a urethane-induced mouse lung cancer model, we demonstrate that early or late intervention with olaparib and hemithoracic radiation enhances inhibition of lung tumour growth without increasing normal lung tissue toxicity, suggesting the potential for an increased therapeutic index.

Radiosensitisation by PARP inhibitors is more pronounced in replicating cells;^31,32^ therefore, PARP inhibition may have greater potential to increase the therapeutic index of radiation when tumours are located in tissues with a low proportion of proliferating cells, such as the lung.^33^ Consistent with this, our recent study in non-tumour-bearing C57BL6 mice^12^ showed that PARP inhibition augmented radiation-induced toxicity in skin and oesophagus, two proliferative normal tissues.^34,35^

Due to our previous work in the C57BL6 strain of mice, we first wanted to confirm the tolerability of olaparib combined with whole thoracic radiation in A/J mice, but we observed significant body weight and oesophageal toxicity. To ameliorate potential normal tissue damage from this combination, we reduced the olaparib to a single dose of 50 mg/kg (which we have previously shown to be an effective radiosensitising treatment in human tumour xenograft studies)^11^ and we also avoided irradiating the oesophagus by using hemithoracic radiation to the left lung. Treatment was initiated at either an early or late stage of lung tumour development. Using this approach, all treatments were well tolerated, and no evident body weight loss or other clinical signs. It has been well established that whole thorax irradiation of both lungs in mice can induce significant histopathological changes (e.g. fibrosis) associated with clear clinical signs of late toxicity (e.g. altered breathing rate, depth) whereas a similar radiation dose delivered to a single lung can induce significant pathology without external clinical signs,^36^ indicating that mice can tolerate substantial damage to a single lung without any apparent physical or behavioural consequences. This is in line with our data presented here where we observed significant lung damage in A/J mice by histology but with no external signs of toxicity following hemithorax irradiation. As anticipated, radiation treatment induced both lung inflammation and fibrosis (collage deposition), but these effects were not increased by combination with olaparib supporting the concept that non-replicating tissues such as lung may be less susceptible to radiosensitisation by olaparib compared with, for example, the oesophagus.

Here, we tested two anti-tumour settings in the A/J model: early and late intervention. Lung tumours are small and undetectable by MRI at an early stage (8 weeks post induction), whereas lung tumours are well developed at a late stage (18 weeks post induction).^19^ By visual scoring, histology and MRI analysis, we found that early intervention with olaparib and left thorax radiation significantly enhanced the reduction in left lung tumour burden compared with radiation alone. In the late intervention study, olaparib also reduced tumour burden compared to radiation alone, but to a lesser extent. These data suggest that early or late intervention with olaparib and hemithoracic radiation can increase the therapeutic index in this mouse model of lung cancer.

Olaparib in combination with thoracic radiation has entered phase 1 clinical trials for lung cancer (NCT01562210, NCT03532880) and phase 1 (NCT02227082, NCT03109080) and phase 2 (NCT03598257) clinical trials for breast cancer. However, the toxicity and efficacy results are still pending for all studies. Our pre-clinical data here provide further supporting evidence for continued evaluation of this combination in the clinic, but if the model translates from mouse to humans, it would suggest that the oesophagus may be sensitive to the combination treatment.

To date, pre-clinical anti-tumour studies combining PARP inhibition and radiation have been predominantly carried out in human subcutaneous xenograft models, which do not mimic many aspects of the tumour microenvironment that could affect responses to treatments.^37^ In addition, subcutaneous xenografts are not suitable for evaluation of normal tissue toxicity as generally only the skin is included in the field of radiation. In contrast, the A/J model recapitulates the many aspects of the natural history of human lung cancer developing in an immune-competent microenvironment, and it allows for concurrent assessment of therapeutic efficacy and both acute and late lung toxicity. Therefore, the A/J model provides a valuable platform to evaluate a clinically relevant therapeutic index, although how these pre-clinical findings translate into the clinic has yet to be determined, although the ongoing clinical trials with olaparib will provide critical information when they report.

Monitoring of lung tumour progression in pre-clinical models is challenging. The recent development of cardio-respiratory synchronised bSSFP MRI enables in vivo quantification of lung tumour growth.^19^ Here, we report for the first time the use of bSSFP MRI to follow tumour response to treatment in vivo. Tumour number and volume determined by the MRI analysis were consistent with visual scoring and histology; therefore, highlighting a potential advantage for MRI in real-time monitoring of lung tumour response to therapy. MRI can also address the potential heterogeneity of treatment response in tumours by identification and measurement of individual tumours at different times.^19^ However, there are limitations of MRI. In particular, some superficial tumours detected by visual scoring are not detected for MRI, presumably because they were below the size limit for detection,^19^ which is why the surface tumour scored visually were numerically greater than those measured by the MRI method used in this study, as we previously reported.^19^

Thoracic radiotherapy-associated oesophagus and lung damage can limit the dose delivery.^38,39^ We showed in the A/J mouse model that oesophageal toxicity was enhanced when olaparib is combined with whole thoracic irradiation but not with hemithoracic radiation. This acute side effect of oesophageal irradiation can be reduced in the clinic by modalities such as conformal planning techniques,^40^ the intensity-modulated radiotherapy or stereotactic radiotherapy^41^ and the symptoms managed, for example, with the use of analgesics and diet modification.^38^ The data in this study indicate that the combination of olaparib and hemithoracic radiotherapy could be a potent therapy for lung cancer in the clinic.

Surprisingly, in animals treated with hemithoracic irradiation, the unirradiated right lungs in the combination group also showed evidence of reduced tumour burden compared with those in the control group. Although of interest, this was an incidental finding in our present work and needs prospective pre-clinical studies to confirm the observation and to understand the underlying biology, which may involve immunological mechanisms, including T cell responses.^42^ A systemic effect on tumours outside the radiation field has been reported in another pre-clinical model, in which two xenografts were implanted at separate sites of the same nude mice. When one tumour was irradiated in combination with capecitabine, the growth of the contralateral unirradiated tumour was also inhibited.^43^ In the clinic, there have been some reports of anti-tumour effects outside the field of irradiation^44–46^ including in lung cancer,^47^ but there is an absence of clinical trial data showing that out-of-field (abscopal) effects contribute to the anti-tumour efficacy of radiation combination therapy.

In conclusion, our study shows that early or late anti-tumour intervention with hemithoracic irradiation plus olaparib enhances anti-tumour effect, but not normal tissue toxicity in a urethane-induced mouse lung cancer model. Our findings provide supporting pre-clinical evidence for the ongoing clinical trials of olaparib in combination with thoracic radiation in lung cancer.

## Supplementary information

Supplementary Figures

## Data Availability

All the datasets for this study are available from the corresponding author on request.

## References

[1] Murai J, Huang SY, Das BB, Renaud A, Zhang Y, Doroshow JH (2012). Trapping of PARP1 and PARP2 by clinical PARP Inhibitors. Cancer Res..

[2] Gelmon KA, Tischkowitz M, Mackay H, Swenerton K, Robidoux A, Tonkin K (2011). Olaparib in patients with recurrent high-grade serous or poorly differentiated ovarian carcinoma or triple-negative breast cancer: a phase 2, multicentre, open-label, non-randomised study. Lancet Oncol..

[3] Laird JH, Lok BH, Ma J, Bell A, de Stanchina E, Poirier JT (2018). Talazoparib is a potent radiosensitizer in small cell lung cancer cell lines and xenografts. Clin. Cancer Res..

[4] Wang L, Mason KA, Ang KK, Buchholz T, Valdecanas D, Mathur A (2012). MK-4827, a PARP-1/-2 inhibitor, strongly enhances response of human lung and breast cancer xenografts to radiation. Invest. N. Drugs.

[5] Senra JM, Telfer BA, Cherry KE, McCrudden CM, Hirst DG, O’Connor MJ (2011). Inhibition of PARP-1 by olaparib (AZD2281) increases the radiosensitivity of a lung tumor xenograft. Mol. Cancer Ther..

[6] Albert JM, Cao C, Kim KW, Willey CD, Geng L, Xiao D (2007). Inhibition of poly(ADP-ribose) polymerase enhances cell death and improves tumor growth delay in irradiated lung cancer models. Clin. Cancer Res..

[7] Donawho CK, Luo Y, Luo Y, Penning TD, Bauch JL, Bouska JJ (2007). ABT-888, an orally active poly(ADP-ribose) polymerase inhibitor that potentiates DNA-damaging agents in preclinical tumor models. Clin. Cancer Res..

[8] Menear KA, Adcock C, Boulter R, Cockcroft XL, Copsey L, Cranston A (2008). 4-[3-(4-cyclopropanecarbonylpiperazine-1-carbonyl)-4-fluorobenzyl]-2H-phthalazin- 1-one: a novel bioavailable inhibitor of poly(ADP-ribose) polymerase-1. J Med Chem.

[9] Michmerhuizen AR, Pesch AM, Moubadder L, Chandler BC, Wilder-Romans K, Cameron M (2019). PARP1 inhibition radiosensitizes models of inflammatory breast cancer to ionizing radiation. Mol. Cancer Ther..

[10] Lee HJ, Yoon C, Schmidt B, Park DJ, Zhang AY, Erkizan HV (2013). Combining PARP-1 inhibition and radiation in Ewing sarcoma results in lethal DNA damage. Mol. Cancer Ther..

[11] Jiang Y, Verbiest T, Devery AM, Bokobza SM, Weber AM, Leszczynska KB (2016). Hypoxia potentiates the radiation-sensitizing effect of olaparib in human non-small cell lung cancer xenografts by contextual synthetic lethality. Int. J. Radiat. Oncol. Biol. Phys..

[12] Lourenco LM, Jiang Y, Drobnitzky N, Green M, Cahill F, Patel A (2018). PARP inhibition combined with thoracic irradiation exacerbates esophageal and skin toxicity in C57BL6 mice. Int. J. Radiat. Oncol. Biol. Phys..

[13] Fong PC, Boss DS, Yap TA, Tutt A, Wu P, Mergui-Roelvink M (2009). Inhibition of poly(ADP-ribose) polymerase in tumors from BRCA mutation carriers. N. Engl. J. Med..

[14] de Haan R, van Werkhoven E, van den Heuvel MM, Peulen HMU, Sonke GS, Elkhuizen P (2019). Study protocols of three parallel phase 1 trials combining radical radiotherapy with the PARP inhibitor olaparib. BMC Cancer.

[15] Westcott PM, Halliwill KD, To MD, Rashid M, Rust AG, Keane TM (2015). The mutational landscapes of genetic and chemical models of Kras-driven lung cancer. Nature.

[16] White MR, Grendon A, Jones HB (1970). Tumor incidence and cellularity in lungs of mice given various dose schedules of urethan. Cancer Res..

[17] Scheenstra AE, Rossi MM, Belderbos JS, Damen EM, Lebesque JV, Sonke JJ (2014). Alpha/beta ratio for normal lung tissue as estimated from lung cancer patients treated with stereotactic body and conventionally fractionated radiation therapy. Int. J. Radiat. Oncol. Biol. Phys..

[18] Williams MV, Denekamp J, Fowler JF (1985). A review of alpha/beta ratios for experimental tumors: implications for clinical studies of altered fractionation. Int. J. Radiat. Oncol. Biol. Phys..

[19] Gomes AL, Kinchesh P, Gilchrist S, Allen PD, Lourenco LM, Ryan AJ (2019). Cardio-respiratory synchronized bSSFP MRI for high throughput in vivo lung tumour quantification. PLoS ONE.

[20] Kersemans V, Gilchrist S, Wallington S, Allen PD, Gomes AL, Dias GM (2019). A carbon-fiber sheet resistor for MR-, CT-, SPECT-, and PET-compatible temperature maintenance in small. Anim. Tomogr..

[21] Kinchesh P, Gilchrist S, Beech JS, Gomes AL, Kersemans V, Newman RG (2018). Prospective gating control for highly efficient cardio-respiratory synchronised short and constant TR MRI in the mouse. Magn. Reson. Imaging.

[22] Yushkevich PA, Piven J, Hazlett HC, Smith RG, Ho S, Gee JC (2006). User-guided 3D active contour segmentation of anatomical structures: significantly improved efficiency and reliability. Neuroimage.

[23] Hubner RH, Gitter W, El Mokhtari NE, Mathiak M, Both M, Bolte H (2008). Standardized quantification of pulmonary fibrosis in histological samples. Biotechniques.

[24] Kirkpatrick, J. P., Soltys, S. G., Lo, S. S., Beal, K., Shrieve, D. C. & Brown, P. D. The radiosurgery fractionation quandary: single fraction or hypofractionation? *Neuro-Oncology* **19**, ii38–ii49 (2017). 10.1093/neuonc/now301 PMC5463582 28380634

[25] Simone CB (2017). Thoracic radiation normal tissue injury. Semin. Radiat. Oncol..

[26] Citrin DE, Shankavaram U, Horton JA, Shield W, Zhao S, Asano H (2013). Role of type II pneumocyte senescence in radiation-induced lung fibrosis. J. Natl Cancer Inst..

[27] Straub JM, New J, Hamilton CD, Lominska C, Shnayder Y, Thomas SM (2015). Radiation-induced fibrosis: mechanisms and implications for therapy. J. Cancer Res. Clin. Oncol..

[28] Abraham DJ, Eckes B, Rajkumar V, Krieg T (2007). New developments in fibroblast and myofibroblast biology: implications for fibrosis and scleroderma. Curr. Rheumatol. Rep..

[29] Gani C, Coackley C, Kumareswaran R, Schutze C, Krause M, Zafarana G (2015). In vivo studies of the PARP inhibitor, AZD-2281, in combination with fractionated radiotherapy: an exploration of the therapeutic ratio. Radiother. Oncol..

[30] Wu M, Liu J, Hu C, Li D, Yang J, Wu Z (2018). Olaparib nanoparticles potentiated radiosensitization effects on lung cancer. Int. J. Nanomed..

[31] Noel G, Godon C, Fernet M, Giocanti N, Megnin-Chanet F, Favaudon V (2006). Radiosensitization by the poly(ADP-ribose) polymerase inhibitor 4-amino-1,8-naphthalimide is specific of the S phase of the cell cycle and involves arrest of DNA synthesis. Mol. Cancer Ther..

[32] Dungey FA, Loser DA, Chalmers AJ (2008). Replication-dependent radiosensitization of human glioma cells by inhibition of poly(ADP-Ribose) polymerase: mechanisms and therapeutic potential. Int. J. Radiat. Oncol. Biol. Phys..

[33] Kauffman SL (1980). Cell proliferation in the mammalian lung. Int. Rev. Exp. Pathol..

[34] Hegazy MA, Fowler JF (1973). Cell population kinetics of plucked and unplucked mouse skin. I. Unirradiated skin. Cell Tissue Kinet..

[35] DeWard AD, Cramer J, Lagasse E (2014). Cellular heterogeneity in the mouse esophagus implicates the presence of a nonquiescent epithelial stem cell population. Cell Rep..

[36] Lockhart SP, Down JD, Steel GG (1992). Mouse hemithoracic irradiation and its interaction with cytotoxic drugs. Radiother. Oncol..

[37] Sharpless NE, Depinho RA (2006). The mighty mouse: genetically engineered mouse models in cancer drug development. Nat. Rev. Drug Discov..

[38] Baker S, Fairchild A (2016). Radiation-induced esophagitis in lung cancer. Lung Cancer.

[39] Vujaskovic Z, Marks LB, Anscher MS (2000). The physical parameters and molecular events associated with radiation-induced lung toxicity. Semin. Radiat. Oncol..

[40] Kong FM, Hayman JA, Griffith KA, Kalemkerian GP, Arenberg D, Lyons S (2006). Final toxicity results of a radiation-dose escalation study in patients with non-small-cell lung cancer (NSCLC): predictors for radiation pneumonitis and fibrosis. Int. J. Radiat. Oncol. Biol. Phys..

[41] Mehta V (2005). Radiation pneumonitis and pulmonary fibrosis in non-small-cell lung cancer: pulmonary function, prediction, and prevention. Int. J. Radiat. Oncol. Biol. Phys..

[42] Rodriguez-Ruiz ME, Vanpouille-Box C, Melero I, Formenti SC, Demaria S (2018). Immunological mechanisms responsible for radiation-induced abscopal effect. Trends Immunol..

[43] Blanquicett C, Saif MW, Buchsbaum DJ, Eloubeidi M, Vickers SM, Chhieng DC (2005). Antitumor efficacy of capecitabine and celecoxib in irradiated and lead-shielded, contralateral human BxPC-3 pancreatic cancer xenografts: clinical implications of abscopal effects. Clin. Cancer Res..

[44] Ohba K, Omagari K, Nakamura T, Ikuno N, Saeki S, Matsuo I (1998). Abscopal regression of hepatocellular carcinoma after radiotherapy for bone metastasis. Gut.

[45] Antoniades J, Brady LW, Lightfoot DA (1977). Lymphangiographic demonstration of the abscopal effect in patients with malignant lymphomas. Int. J. Radiat. Oncol. Biol. Phys..

[46] Rees GJ, Ross CM (1983). Abscopal regression following radiotherapy for adenocarcinoma. Br. J. Radiol..

[47] Bitran J (2019). The abscopal effect exists in non-small cell lung cancer: a case report and review of the literature. Cureus.

