# Supplementary material for: Olaparib increases the therapeutic index of hemithoracic irradiation compared with hemithoracic irradiation alone in a mouse lung cancer model
Source: Br J Cancer. 2021 Mar 19;124(11):1809–19. doi: 10.1038/s41416-021-01296-y (PMC8144220; doi:10.1038/s41416-021-01296-y)
Supplement: Supplementary file 1 — Supplementary Figures [file 41416_2021_1296_MOESM1_ESM.pptx]

## Slide 1
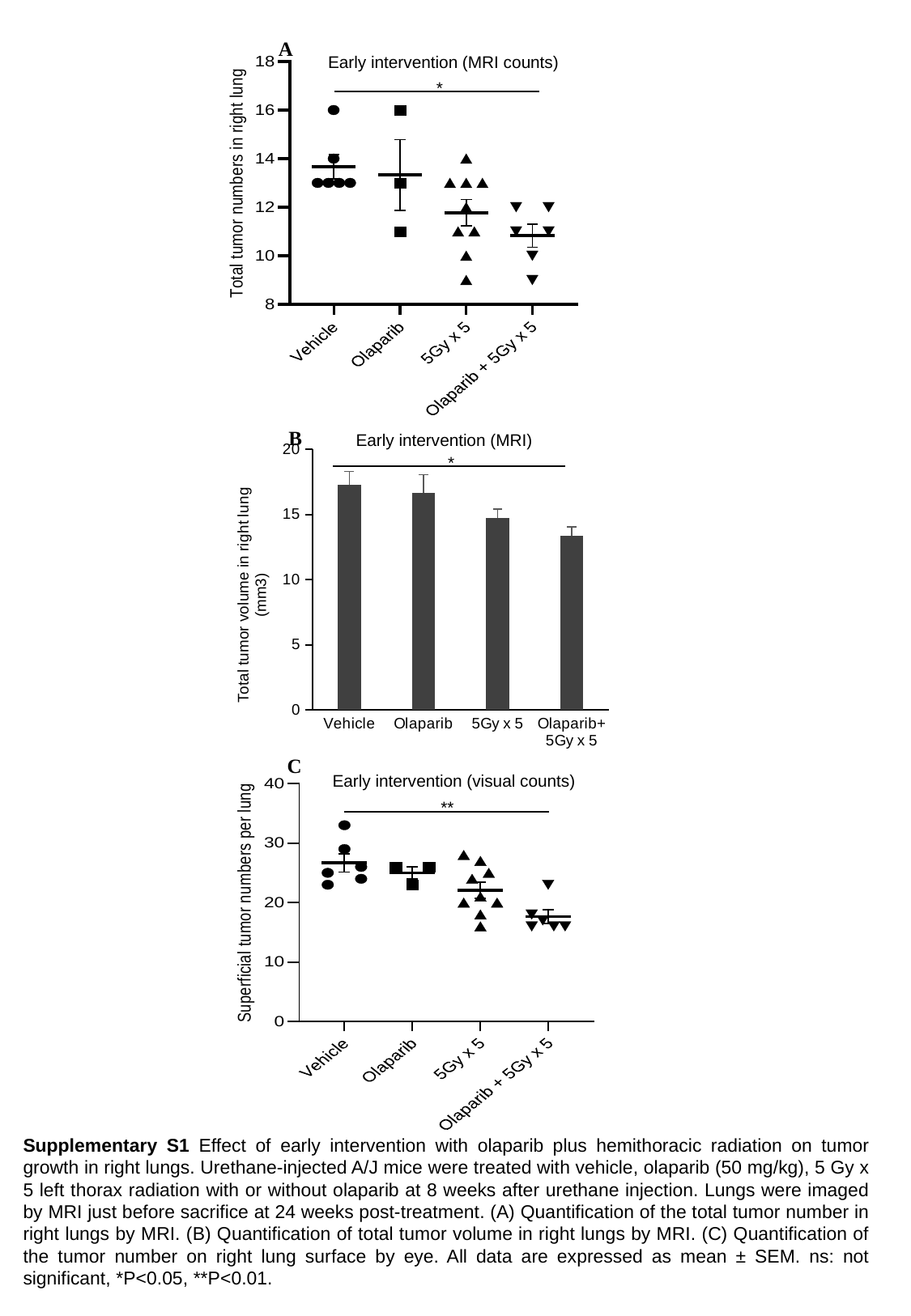

A
Early intervention (MRI counts)
*
B
Early intervention (MRI)
### Chart
| Category | |
|---|---|
| Vehicle | 17.25 |
| Olaparib | 16.68 |
| 5Gy x 5 | 14.71 |
| Olaparib+ 5Gy x 5 | 13.38 |*
C
Early intervention (visual counts)
**
Supplementary S1 Effect of early intervention with olaparib plus hemithoracic radiation on tumor growth in right lungs. Urethane-injected A/J mice were treated with vehicle, olaparib (50 mg/kg), 5 Gy x 5 left thorax radiation with or without olaparib at 8 weeks after urethane injection. Lungs were imaged by MRI just before sacrifice at 24 weeks post-treatment. (A) Quantification of the total tumor number in right lungs by MRI. (B) Quantification of total tumor volume in right lungs by MRI. (C) Quantification of the tumor number on right lung surface by eye. All data are expressed as mean ± SEM. ns: not significant, *P<0.05, **P<0.01.

## Slide 2
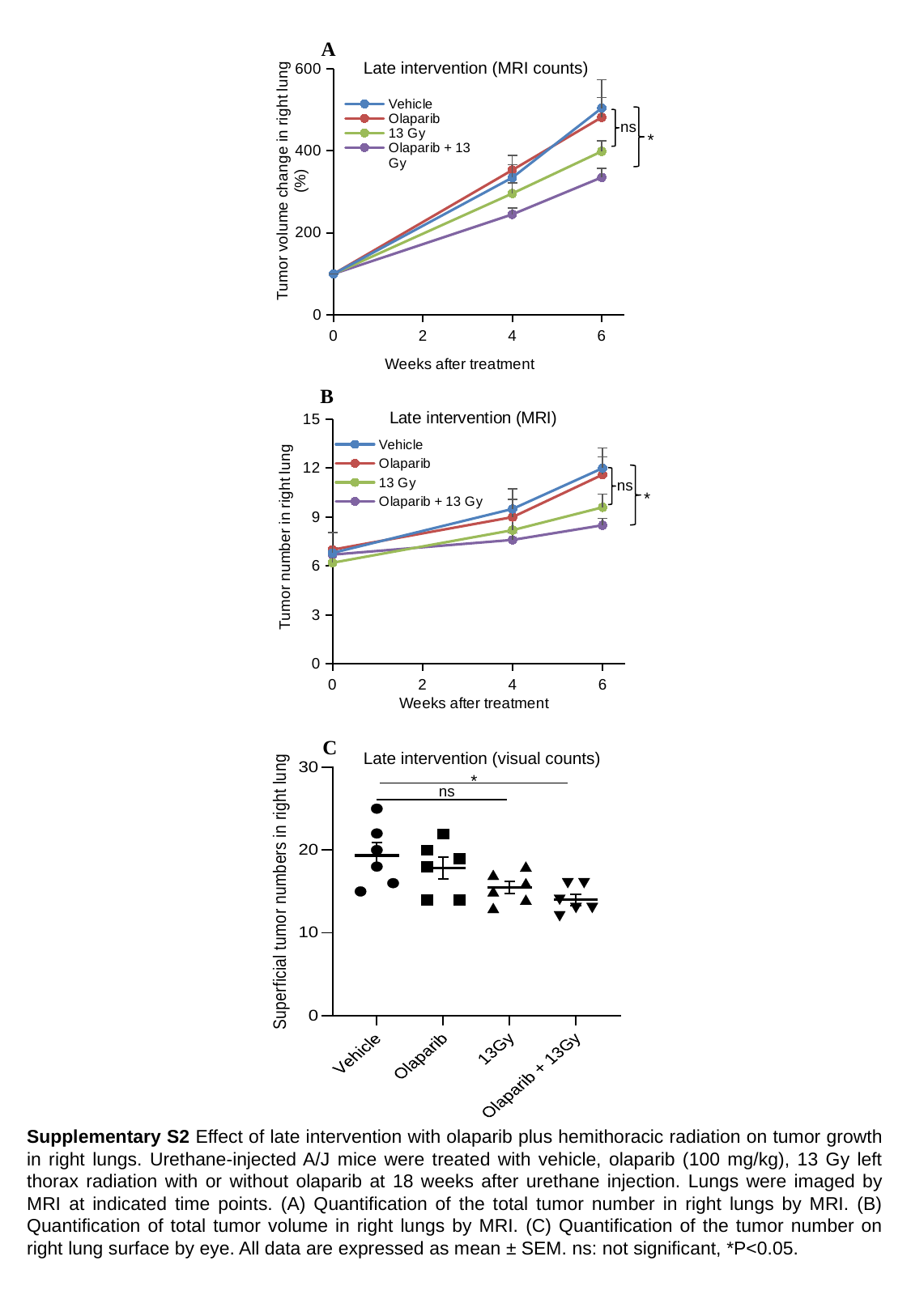

A
Late intervention (MRI counts)
### Chart
| Category | Vehicle | Olaparib | 13 Gy | Olaparib + 13 Gy |
|---|---|---|---|---|
ns
*
B
### Chart
| Category | Vehicle | Olaparib | 13 Gy | Olaparib + 13 Gy |
|---|---|---|---|---|
ns
*
C
Late intervention (visual counts)
*
ns
Supplementary S2 Effect of late intervention with olaparib plus hemithoracic radiation on tumor growth in right lungs. Urethane-injected A/J mice were treated with vehicle, olaparib (100 mg/kg), 13 Gy left thorax radiation with or without olaparib at 18 weeks after urethane injection. Lungs were imaged by MRI at indicated time points. (A) Quantification of the total tumor number in right lungs by MRI. (B) Quantification of total tumor volume in right lungs by MRI. (C) Quantification of the tumor number on right lung surface by eye. All data are expressed as mean ± SEM. ns: not significant, *P<0.05.
